# Effects of Medicinal Plants and Organic Selenium against Ovine Haemonchosis

**DOI:** 10.3390/ani11051319

**Published:** 2021-05-05

**Authors:** Michaela Komáromyová, Dominika Mravčáková, Daniel Petrič, Katarína Kucková, Michal Babják, Michaela Urda Dolinská, Alžbeta Königová, Michaela Maďarová, Ewa Pruszyńska-Oszmałek, Adam Cieslak, Klaudia Čobanová, Zora Váradyová, Marián Várady

**Affiliations:** 1 Institute of Parasitology, Slovak Academy of Sciences, Hlinkova 3, 040 01 Košice, Slovakia; komaromyova@saske.sk (M.K.); babjak@saske.sk (M.B.); dolinska@saske.sk (M.U.D.); konig@saske.sk (A.K.); 2 University of Veterinary Medicine and Pharmacy in Košice, Komenského 73, 041 81 Košice, Slovakia; 3 Centre of Biosciences of Slovak Academy of Sciences, Institute of Animal Physiology, Šoltésovej 4-6, 040 01 Košice, Slovakia; mravcakova@saske.sk (D.M.); petric@saske.sk (D.P.); kuckova@saske.sk (K.K.); mad.michaela@gmail.com (M.M.); boldik@saske.sk (K.Č.); 4 Biochemistry and Biostructure, Department of Animal Physiology, Poznan University of Life Sciences, Wolynska 33, 60-637 Poznan, Poland; ewa.pruszynska@up.poznan.pl; 5 Department of Animal Nutrition, Poznan University of Life Sciences, Wolynska 33, 60-637 Poznan, Poland; adam.cieslak@up.poznan.pl

**Keywords:** *Haemonchus contortus*, Se-yeast, plant nutraceuticals, vitamin E, antioxidants, parasitological status, thyroxine, triiodothyronine

## Abstract

**Simple Summary:**

Maintaining high productivity in small ruminants is hindered by infection with the parasitic gastrointestinal nematode (GIN) *Haemonchus contortus*. There is an increasing interest to find alternative treatments for controlling GINs due to the rapid development of resistance to synthetic anthelmintics. Supplementary feeding of plant nutraceuticals or mineral supplements may contribute to improving the resilience of lambs. The trace element selenium and vitamin E act synergistically as antioxidants in circulation, but the natural occurrence of selenium is very low in the soil, grain, and roughage. The aim of this study was therefore to determine the long-term effects of medicinal plants and organic selenium in lambs experimentally infected with *H. contortus*. The results indicated that both supplements favorably affected the infected lambs and may provide a new approach to controlling haemonchosis in small ruminants.

**Abstract:**

The objective of this study was to determine the effect of traditional medicinal plants typical to Central Europe as well as organic selenium on increasing the resistance of lambs to gastrointestinal nematode (GIN) infection with *Haemonchus contortus.* 21 female lambs were infected with third-stage larvae of *H. contortus* on the day (D) 0 and re-infected on D49 and D77. The animals were divided into three groups based on a treatment diet: a basal diet (control), a diet enriched with dry plants (Herbmix), and a diet enriched with selenized yeast (Selplex). The number of eggs per gram (EPG) of feces was quantified on D21, D28, D35, D42, D49, D56, D63, D70, D77, D84, D91, D98, D105, D112, and D119. The mean reductions in EPG on D28 were 43.4 and 28.6% for Selplex and Herbmix, respectively. The reduction in egg output was nearly uniform throughout the experiment for Selplex. However, for Herbmix the mean reduction was only 19.8% up to D91 and 46.1% after D91. Glutathione peroxidase activity in the blood from D35 to D98 was two to three-fold higher for Selplex than the other groups. Both supplements slowed the dynamics of GIN infection and gradually increased the resistance of lambs against ovine haemonchosis.

## 1. Introduction

Anthelmintic strategies for controlling gastrointestinal nematode (GIN) parasites in small ruminants mostly use the repeated application of synthetic drugs that can lead to the development of anthelmintic resistance [1,2]. The current global trend is to develop new anthelmintics that are effective mainly against resistant species of nematodes [3]. Well-known anthelmintic effects of herbal medicines against the GIN *Haemonchus contortus* are associated with bioactive compounds, mainly tannins, saponins, and flavonoids, with two possible general mechanisms of action: direct effects on the parasite, or indirect effects by interaction with the host immune system [4]. The scientific evaluation of plant nutraceuticals against GIN parasites (similar to those developed to assess the efficacy of synthetic drugs against GINs) however, requires a multidisciplinary approach between parasitologists and other scientists [5,6]. Our previous studies found that the effect of medicinal plants on GIN-infected sheep depended on the variety and synergy of polyphenols in medicinal plants and on the combination of bioactive compounds that together had an effect contributing to pharmacological efficacy [7,8,9,10]. Most studies conducted mainly with the use of medicinal plants in ruminants, however, have generally found lower effectiveness of plant materials against parasites *in vivo* compared to *in vitro* [11,12]. The presence of a GIN infection also disturbs the mineral metabolism of ruminants, and mineral deficiencies in nutrition are associated with increased GIN burdens in animals. The production of reactive oxygen species by host phagocytes (macrophages, eosinophils, and neutrophils) plays an important role in the ability of hosts to expel GIN parasites [13]. These reactive oxygen species cause immunosuppression by intensive oxidative processes [14]. These processes, however, can be neutralized by an antioxidant defensive system represented by enzymes such as catalase, superoxide dismutase, glutathione peroxidase (GPx) and by nonenzymatic antioxidants such as vitamins A, C, and E [15]. Some trace elements (e.g. copper and zinc) in feed supplements can reduce oxidative stress and increase resistance against *H. contortus* infection in lambs [16,17]. The trace element selenium (Se) is also an integral part of the antioxidant enzyme GPx and is important for the adequate functioning of the immune system [18,19]. Se concentration in blood has been strongly correlated with the activity of GPx [19].

Natural levels of Se in soil, grain, and roughage are very low in most countries [20,21], and the statuses of Se and vitamin E are closely linked with the status of antioxidants in animals [22]. The antioxidant functions of Se and vitamin E are interdependent, so Se deficiency can be partially compensated by an adequate intake of vitamin E and *vice versa* [23]. Se, particularly in synergism with vitamin E, prevents the oxidation of polyunsaturated fatty acids in membranes and of DNA by oxygen radicals produced throughout aerobic metabolism [24]. Se is also essential for the functioning of thyroid hormones; the selenoenzyme iodothyronine deiodinase is required for the deiodination of the thyroid hormone thyroxine (T4) to the more metabolically active triiodothyronine (T3) [25]. Limited information, however, is available on the effects of natural dietary additives on serum concentrations of vitamin E and hormones in sheep with parasitic GIN infections [26].

Supplementing diets with organic Se has been hypothesized to also provide better protection against haemonchosis in lambs. Our previous studies found a beneficial effect of selecting and combining traditional dry medicinal plants in the diet of lambs with haemonchosis [7,8,9,10], but we only monitored this effect in the short term (60–75 d). The effect of the long-term application of medicinal plants and organic Se as dietary supplements on T3 and T4 during haemonchosis of sheep, however, has not yet been described. The objective of this study was therefore to determine the effect of a mixture of dry medicinal plants (Herbmix) and organic Se (Selplex) on live-weight gain, parasitological status, hematological parameters, antioxidant status, serum concentration of vitamin E, and T3 and T4 concentrations over a longer period (119 d) in lambs experimentally infected and re-infected with *H. contortus*.

## 2. Materials and Methods

### 2.1. Ethics Statement

This study was conducted following the guidelines of the Declaration of Helsinki and national legislation in the Slovak Republic (G.R. 377/2012; Law 39/2007) for the care and use of research animals. The experimental protocol was approved by the Ethical Committee of the Institute of Parasitology of the Slovak Academy of Sciences on 14 October 2019 (protocol code 2019/17).

### 2.2. Animals, Diets, and Experimental Design

We housed 21 female lambs (Improved Valachian), 3–4 months of age, with an average initial body weight of 18.41 ± 0.43 kg, in common stalls for 14 d for acclimatizing to feeding, with free access to water. The lambs were obtained from a commercial farm (PD Ružín–Ružín farm, Kysak, Slovakia) where they also were housed during the experiment. Each animal was fed meadow hay (*ad libitum*) and 300 g dry matter (DM)/d of Mikrop ČOJ, a commercial concentrate (MIKROP, Čebín, Czech Republic). All parasite-free lambs were then separated into three stalls and infected orally with approximately 5000 third-stage larvae of the MHCo1 strain of *H. contortus*, that is susceptible to anthelmintics [8]. The number of animals used in the experiment was assigned following VICH GL13 guidelines (Veterinary International Committee on Harmonization - Efficacy of anthelmintics: specific requirements for ovines). Lambs were randomly divided by live weight into three groups of seven animals each (one stall per group): unsupplemented animals (control), animals supplemented with dry medicinal plants (Herbmix, 100 g DM/d/animal), and animals supplemented with organic Se (Selplex). Aliquots of the Selplex (selenized yeast, SEL-PLEX 2300; Alltech, Nitra, Slovakia) were directly mixed with the commercial concentrate at the amount of 0.8 mg Se/kg concentrate to provide the additional 0.24 mg Se/animal. The upper limit of Se allowed in feed is 0.5 mg/kg complete feed for all species of animals [27]. The Herbmix was obtained from commercial sources (AGROKARPATY, Plavnica, Slovak Republic and BYLINY Mikeš s.r.o., Číčenice, Czech Republic). The Herbmix contained a mixture of nine medicinal plants (i.e., *Althaea officinalis*, *Petasites hybridus*, *Inula helenium*, *Plantago lanceolata*, *Rosmarinus officinalis*, *Foeniculum vulgare*, *Solidago virgaurea*, *Fumaria officinalis*, and *Hyssopus officinalis*). The phytochemical substances flavonoids (9.965 g/kg), diterpenes (4.886 g/kg), and phenolic acids (3.549 g/kg) with high concentrations of quercetin-O-Hex-dHex (1.44 g/kg DM), verbascoside (1.16 g/kg DM), 3,5-dicaffeoyl-quinic acid (1.60 g/kg DM), quercetin-O-dHex-dHex (1.44 g/kg DM), rosmarinic acid (2.89 g/kg DM), and carnosol (1.36 g/kg DM) [8]. Herbmix and Selplex supplementation began on day (D) 0. The lambs of all groups were re-infected on D49 and D77 with approximately 3000 third-stage larvae. The experimental period was 119 d. All animals were killed at the end of the experiment following the rules of the European Commission (Council Regulation 1099/2009) for slaughtering procedures [28].

### 2.3. Chemical Composition of Dietary Substrates

The chemical compositions of the dietary substrates (Table 1) were analyzed using standard methods [29,30].

Chemical tests for the screening of phytochemicals were carried out in aqueous extracts (saponins, terpenoids) or ethanolic extracts (tannins, alkaloids, flavonoids, steroids) (Table 2) using standard procedures [31].

### 2.4. Animal Weighing and Parasitological Analyses

The lambs were weighed on D0, D35, D63, and D98. Feces were collected from the rectum, and the number of eggs per gram (EPG) of feces was quantified on D21, D28, D35, D42, D49, D56, D63, D70, D77, D84, D91, D98, D105, D112, and D119 as previously described [8]. The number of *H. contortus* eggs was determined using the McMaster technique [32]. The abomasum of each lamb (slaughtered on D119) was removed and dissected, and the contents were emptied into a bucket. The abomasal mucosa was washed gently with water, washing the parasites into the bucket. The contents of the bucket were adjusted to two liters and thoroughly mixed. Two 100-mL aliquots were then taken, and the number of *H. contortus* in each aliquot was counted. 

### 2.5. Hematological Analyses

Samples of blood were collected from each animal on D0, D15, D35, D49, D63, D77, D98, and D112. Basic hematological parameters were determined using an Abbott CELL-DYN 3700 hematological analyzer (Global Medical Instrumentation, Inc., Ramsey, NJ, USA). For the analysis of vitamin E, 0.6 mL of blood serum was deproteinized with 0.6 mL of methanol and then extracted with 0.6 mL of n-hexane. The extract was vortexed for 3 min and centrifuged (approximately 1200× *g* for 8 min). 500 microliters of the organic phase were then collected and evaporated to dryness under nitrogen. The residue was dissolved in 100 µL of methanol (Merck KGaA, Darmstadt, Germany). The sample was prepared for injection onto a chromatographic column, and analytes were detected using an external standard calibration. Each sample was measured three times. Chromatographic analysis was performed on a 1260 Infinity liquid chromatographic system (Agilent Technologies, Santa Clara, CA, USA) consisting of a G1311C 1260 Quaternary Pump, a Pump VL, a G1367E 1260 Hip autosampler, an ALS, a G1314F 1260 VWD UV/VIS detector, and a G1316C 1290 TCC column thermostat. Vitamin E was separated on a Phenomenex Jupiter 5u C18 300A column (250 × 4.6 mm, 5 μm). Methanol (100%) was used as the mobile phase at a flow rate of 1 mL/min. UV detection of α-tocopherol was performed at 292 nm at a sample injection of 20 µL at room temperature. Vitamin E was eluted at a retention time of 6.6 min. The total analysis time was 8 min. Quantification was performed based on a calibration curve for an external standard. Samples were quantified using the area under the α-tocopherol peak. A standard calibration curve was constructed with a known amount of vitamin E. The linearity of the calibration curve for α-tocopherol was obtained at six concentrations ranging from 0.05 to 30.0 mg/L based on peak area (r = 0.99993).

The activity of GPx in the total blood, total antioxidant capacity in the serum, and the serum concentration of malondialdehyde (MDA) were determined as previously described [17].

Radioimmunoassay kits (Cat. Nos. MG13081 and MG13091; IBL International GmbH, Hamburg, Germany) were used to determine the concentrations of T3 and T4, respectively, in the blood sera of the sheep [33,34,35]. All procedures were performed following the instructions provided by the manufacturers. The radioactivity of the samples was measured using a Wizard2 2-Detector Gamma Counter (Perkin Elmer, Waltham, MA, USA).

### 2.6. Statistical Analysis 

Statistical analysis was performed using analyses of variance (ANOVAs) (GraphPad Prism 8; GraphPad Software, Inc., San Diego, CA, USA) as repeated-measures mixed models representing the three animal groups (control, Herbmix, and Selplex) and sampling days (D0-D119). The effects included in the model were treatment (T), time, and the interaction between treatment and time (T × Time). Differences between the groups were identified using a two-way ANOVA with a Bonferroni *post* hoc test. Student’s *t*-tests were applied to assess the differences between mean egg outputs (EPGs) on different sampling days and worm counts at dissection. Results were considered significant at *p* < 0.05.

## 3. Results

### 3.1. Weight and Parasitological Status

Mean body weights (BWs) and live-weight gains (LWGs) did not differ significantly (*p* > 0.05) between the control, Herbmix, and Selplex groups (Table 3). Only time affected BW and the mean cumulative LWG (*p* < 0.001).

**Figure 1:**
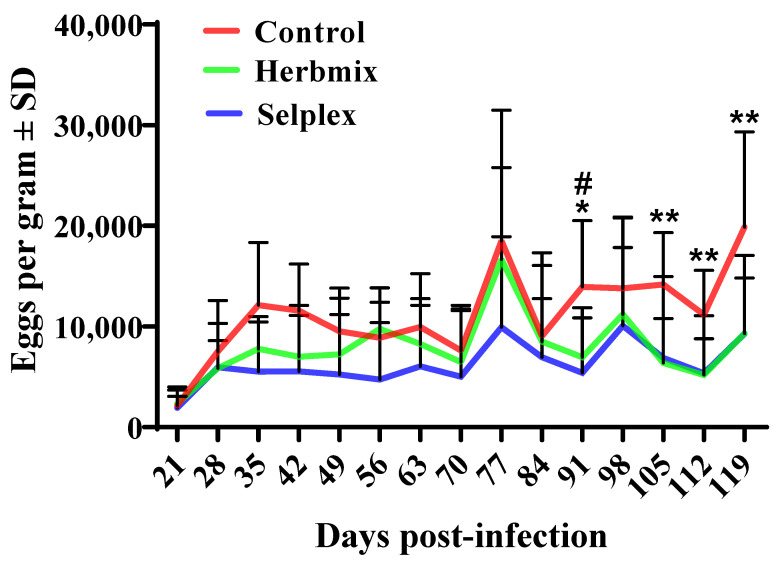
Fecal egg counts (mean ± SD) for the groups of lambs infected with *H. contortus.* *^,^ ** Herbmix and # Selpex group differed significantly from control group (*p* < 0.05 and *p* < 0.01). The re-infection was on D49 and D77.

The patterns of egg shedding for the control, Herbmix, and Selplex groups are shown in Figure 1. The data from D21 were compared and used to determine the reduction in egg output for Herbmix and Selplex relative to the control. EPGs were influenced by treatment (*p* < 0.001) and time (*p* < 0.001). Treatment and time did not significantly interact (*p* > 0.05). The egg output on D91 was significantly lower (*p* < 0.05) for Herbmix and Selplex than the control. The egg counts after D105 were also significantly lower for Herbmix than the control (*p* < 0.01). The mean reductions in egg output on D28 were 43.4 and 28.6% for Selplex and Herbmix, respectively. The reduction in egg output was nearly uniform throughout the experiment for Selplex. However, for Herbmix the mean reduction was only 19.8% up to D91 and 46.1% after D91. 

**Figure 2:**
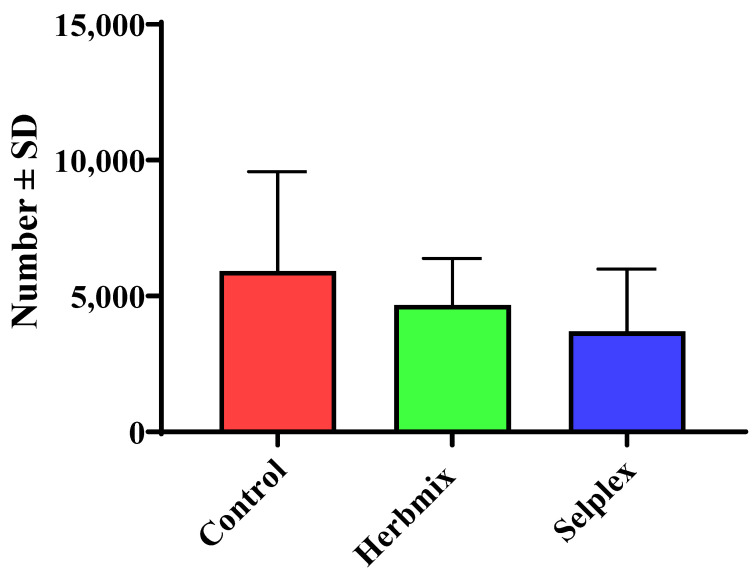
Abomasal worm counts (number ± SD) of *H. contortus* in the lambs of each treatment at the end of the experiment on day 119 (*p* > 0.05).

The abomasal worm counts in the lambs treated with Herbmix and Selplex did not differ significantly from the count in the control (*p* > 0.05). The abomasal worm counts at necropsy on D119 were lower for Herbmix and Selplex than the control, but not significantly (*p* > 0.05) (Figure 2).

### 3.2. Red Blood Cell Hemograms

The complete red blood cell (RBC) hemograms of each infected animal identified clinical signs of haemonchosis such as anemia from D35 (Table 4). Anemia, as revealed by a decrease of red blood cell, hemoglobin and hematocrit concentrations, was observed in all infected groups, and that was settled until the end of the experiment. The RBC count, hemoglobin (HGB) level, hematocrit (HCT), and mean corpuscular volume (MCV) were influenced by time (*p* < 0.001). RBC count (*p* = 0.003) and MCV (*p* < 0.001) were also influenced by treatment. 

### 3.3. Vitamin E 

The concentration of vitamin E in the sera of the infected lambs was influenced by treatment (*p* < 0.001), time (*p* < 0.001), and the T × Time interaction (*p* = 0.002) (Table 5).

### 3.4. Antioxidant Status 

Treatment, time, and the interaction between treatment and time affected GPx activity (*p* < 0.001) (Table 6). GPx activity from D35 to D98 was two- to three-fold higher for Selplex than the other groups. The total antioxidant capacity and serum MDA concentration were significantly influenced by time in all groups (*p* < 0.001).

### 3.5. Triiodothyronine and Thyroxine 

The concentration of T3 in the blood sera was influenced by treatment and the interaction between treatment and time (*p* < 0.001 and *p* = 0.021, respectively), and the concentration of T4 was influenced by treatment and time (*p* = 0.047 and *p* = 0.020, respectively) (Table 7). The T3:T4 ratio was affected by treatment and the interaction between treatment and time (*p* < 0.001 and *p* < 0.004, respectively). The concentrations of both T3 and T4 were highest for Selplex. The concentration of T3, however, was 2.5-fold higher for Selplex than the control and Herbmix on D35 and D63 and >20-fold higher on D98.

## 4. Discussion

Natural anthelmintics and dewormers have been studied for many decades, but more than three dozen anthelmintic compounds have been isolated from medicinal plants in the last 15 years, most of which have been used traditionally to treat gastrointestinal nematodes [36]. The medicinal plants we used belong mainly to the families Asteraceae (*P. hybridus*, *I. helenium*, and *S. virgaurea*) and Lamiaceae (*R. officinalis* and *H. officinalis*). We have previously described the main bioactive compounds in the plants and their mixtures [8,11]. The findings from European studies of ethnoveterinary medicine indicate that plants from the Asteraceae, Lamiaceae, and Fabaceae families are especially promising for treating gastrointestinal disorders and parasitosis [37].

We could not confirm the effect of Herbmix on BW and LWG in the infected lambs, similarly to our previous experiment [8]. Another mixture containing 13 medicinal plants (the nine in our study plus *Artemisia absinthium*, *Melissa officinalis*, *Matricaria chamomilla*, and *Malva sylvestris*), however, significantly affected the daily LWG of infected lambs (44 g/d untreated *versus* 108.4 g/d treated lambs) [7]. Weight gain in sheep infected with *H. contortus* is about 77% of the gain in parasite-free animals, but GINs in a meta-analysis significantly negatively affected animal production in only 58.3% of trials [38]. Dietary supplementation with *A. absinthium* for 90 d can increase the rate of growth and weight gains of lambs [39]. An extract of *M. chamomilla* (5 mL/kg BW) used as a dewormer significantly increased the BWs of lambs [40]. Herbmix in our study contained neither *A. absinthium* nor *M. chamomilla*, so adding dry medicinal plants to the diets of GIN-infected lambs may or may not have influenced BW or LWG [7,8,9,10]. Phenolic compounds, especially flavonoids, however, can generally improve LWG, the growth of animals, and the quality of animal products [41].

Mean egg output did not differ significantly among the three infected groups up to D77. The relatively high SDs of the means for the treated groups throughout the experiment, however, indicated a potentially different effect of treatment between lambs. We also observed similarly high SDs of the means in our previous experiments [9,10]. Egg production remained lower (*p* *>* 0.05) for both treated groups than the control from D28 to D56 (approximately 5400 and 7500 EPG for Selplex and Herbmix, respectively) and then decreased more. We previously reported a similar beneficial effect of mixtures of medicinal plants between D35 and D50 in infected animals [7,8,9,10]. The mean reduction in egg production between the control and treated lambs in our study was not significant on most days, but the mean reduction in fecal egg counts by almost 50% would substantially reduce pasture infectivity, which would certainly have epidemiological benefits for sheep on pasture. Unlike our previous studies, however, the lambs were re-infected twice to simulate natural pastoral conditions. The high egg production on D77 in all groups could be attributed to re-infection on D49. EPG was similarly higher on D98 in both treated groups in response to a second re-infection on D77. Interestingly, EPGs remained high in the control until the end of the experiment. Egg output in the Selplex group throughout the experiment and in the Herbmix group after D91 rapidly decreased relative to the control, indicated by fewer adult *H. contortus* in both treated groups. The EPG and worm burden of lambs experimentally infected with *H. contortus* decreased significantly (*p* < 0.05) in a study that injected a Se supplement (0.2 mg/kg sodium selenite) alone or with copper [42]. Se levels in blood under normal circumstances are highest (0.25–0.31 mg Se/kg BW) from 60 to 80 d after injection [43]. The differences between the results obtained in dissection and EPG values in egg counts between treated and control groups could be explained by the fact that female of *H. contortus* parasites may suppress the egg excretion. This phenomenon of temporary or permanent suppression often occurs after treatment with either anthelmintic or nutraceuticals.

Our study confirmed a marked reduction in RBC, HGB, and HCT from D35 of the experiment in all three experimental groups due to the damage caused by the parasites. Previous studies of GIN-infected lambs have reported similar results [7,8]. Treatment with Herbmix or organic Se in our study only slightly improved the RBC parameters, even though the intensity and length of hematological disturbances depend on the nutritional status of sheep with haemonchosis [44]. Treatment with the anthelmintic moxidectin and maize dietary supplementation can return RBC parameters in sheep with haemonchosis to normal levels [45]. Clinically differentiating haemonchosis caused by *H. contortus* from malnutrition, however, is difficult, because both are common in many production systems [46]. The MCV of the RBCs in our study increased regardless of dietary status and was the highest in the Herbmix group, consistent with our previous results with infected lambs [7]. A similar increase in MCV was reported for Creole goats infected with *H. contortus* fed four diets with different contents of protein and energy [44]. Clinical signs of haemonchosis such as anemia have been correlated with clinicopathological and parasitological findings and are helpful for detecting and diagnosing *H. contortus* infections in sheep and goats [47]. 

Colostrum is the main source of vitamin E for newborn lambs, but early food diversification may restore a deficient supply of vitamin E [48]. Limited information is available on the effect of GIN parasites on serum concentrations of vitamin E in lambs, but these parasites can disrupt the absorption and retention of vitamins [49,50]. The serum concentration of tocopherol was affected by the interaction between treatment and time in our study, so the relationship can be presumed to be between the patent period of *H. contortus* and the treatment of the lambs. GIN infection in a previous study did not significantly affect the serum concentration of vitamin E [26], which probably depended on the difference between the level of parasitism and the potential of the treatments to enhance resistance to GIN infection in growing lambs, consistent with our results. Vitamin E supplementation (15 and 30 IU/kg BW) for 28 weeks, however, increased serum tocopherol concentration by approximately 30% but did not affect parasitological parameters (EPG, HCT, and GIN burden) in pasture-infected lambs [51]. 

Both vitamin E and Se can protect cellular membranes from oxidative degeneration [52]. Our results indicated that the activity of GPx (a Se-dependent antioxidant enzyme) in the total blood was highest in the Selplex group throughout the experiment. Our results also indicated that lambs in the control and Herbmix groups infected with *H. contortus* could have Se deficiency, which would decrease GPx activity relative to the Selplex group, thereby probably also increasing the vulnerability of cells to the harmful actions of reactive oxygen species [53]. Se can reduce the effects of oxidative stress caused by infection with *H. contortus* and can also reduce EPG and the number of adult parasites in association with another trace element, copper [14]. This activity of Se was apparent throughout our experiment on EPGs and the reduction in worm burdens after necropsy. 

The concentrations of both T3 and T4 and the T3:T4 ratio differed between the treatments. In contrast, the supplementation of lambs with sodium selenite (0.2 ppm) for 19 weeks only slightly increased total plasma T3 concentration (*p* < 0.05 for 5, 7, 9, and 10 weeks), but the T4 concentration and the T3:T4 ratio were not affected [33]. The levels of both hormones during infection in our experiment were highest in the Selplex group. GIN infections may also affect the absorption and retention of minerals [50,54]. T4 concentrations in the control and Herbmix groups were relatively stable during infection, but the concentration of the more potent T3 was relatively stable only on D35 and D63 and had decreased rapidly by D98. These data suggest that lambs infected with *H. contortus* exhibited Se deficiency, and the effect was not due to impaired conversion of T4 to T3. Also, supplementation with Se in the Selplex group indicated that deiodinase activity was homeostatically controlled, and thus Se was likely incorporated for the maintenance of thyroid hormone homeostasis [33,55]. Sheep are also likely to be more resistant to the effects of Se deficiency on the metabolism of thyroid hormones than rats, humans, or cattle [56]. The organic form of Se mainly contributes to maintaining a physiological concentration of Se, preventing thyroid disease, and preserving overall health, with benefits that improve immunological mechanisms [57].

## 5. Conclusions

This study confirmed the beneficial effect of medicinal plants based on their variety, synergy, and combination of bioactive compounds against haemonchosis in lambs. Our study also found that the beneficial effect of organic Se protected immune cells from damage induced by oxidative stress caused by infection with *H. contortus*. Both the plants and Se provided by supplementation probably indirectly contributed to an increase in host resistance by improving immunocompetence to nematode infection and a sustainable reduction in pasture contamination by reduced egg output from infected lambs.

## Figures and Tables

**Table 1:** Chemical compositions of the dietary substrates.

Dietary Substrate	DM (g/kg)	NDF (g/kg DM)	ADF (g/kg DM)	CP (g/kg DM)	N (g/kg DM)	Ash (g/kg DM)
Meadow hay	917	675	445	74	12	68
Concentrate	899	263	145	261	42	124
Selplex	898	238	137	262	42	146
Herbmix	908	346	326	161	26	116

DM, dry matter; NDF, neutral detergent fiber; ADF, acidic detergent fiber; CP, crude protein; N, nitrogen.

**Table 2:** Chemical compositions of the aqueous and ethanolic herbal extracts.

Plant Species	Tannins	Saponins	Alkaloids	Terpenoids	Flavonoids	Steroids
*Althaea officinalis*	−	−	−	+	+	−
*Petasites hybridus*	−	+	−	−	−	+
*Inula helenium*	−	+	−	+	−	+
*Plantago lanceolate*	+	−	−	+	−	+
*Rosmarinus officinalis*	+	−	+	−	+	+
*Foeniculum vulgare*	−	−	−	+	−	+
*Solidago virgaurea*	+	+	−	−	−	+
*Fumaria officinalis*	+	−	−	+	+	−
*Hyssopus officinalis*	−	−	+	−	−	−
Herbmix	+	+	+	+	+	+

+, phytochemicals present; −, phytochemicals not present.

**Table 3:** Body weight (BW) and mean live-weight gain (LWG) of the experimental lambs (*n* = 7).

	Day	Control	Herbmix	Selplex	SD	Significance of Effects (*p*-Value)		
						T	Time	T × Time
BW (kg)	0	18.7	18.1	18.8	2.26	0.559	0.001	0.999
	35	22.7	22.3	22.4	2.74			
	63	25.5	24.9	24.7	2.96			
	98	27.4	26.9	26.5	3.15			
LWG (kg)	35	4.09	4.21	3.59	0.792	0.613	0.001	0.494
	63	2.81	2.60	2.34	0.742			
	98	1.86	1.93	1.73	1.075			

T, treatment; SD, standard deviation.

**Table 4:** Hematological parameters of the experimental lambs (*n* = 7).

Item	Day	Control	Herbmix	Selplex	SD	Significance of Effects (*p*-Value)		
						T	Time	T × Time
RBC	0	12.1	12.3	12.5	0.68	0.003	0.001	1.000
(T/L)	15	11.4	11.4	12.0	0.88			
	35	9.18	9.17	10.2	1.31			
	49	8.57	8.38	9.53	1.32			
	63	8.24	8.40	9.43	1.41			
	77	7.83	8.08	8.93	1.41			
	98	7.04	6.98	7.78	1.84			
	112	6.91	7.14	7.62	1.91			
HGB	0	125	120	120	8.47	0.088	0.001	0.977
(g/L)	15	110	109	113	7.01			
	35	90.3	92.9	95.8	9.92			
	49	87.9	88.1	89.5	11.7			
	63	80.8	86.1	87.2	14.9			
	77	79.1	83.5	85.2	14.3			
	98	69.3	71.4	74.5	20.7			
	112	63.6	70.5	73.6	22.3			
HCT	0	0.255	0.262	0.247	0.013	0.197	0.001	1.000
(g/L)	15	0.240	0.243	0.237	0.011			
	35	0.216	0.226	0.216	0.018			
	49	0.213	0.216	0.206	0.023			
	63	0.198	0.214	0.204	0.030			
	77	0.197	0.210	0.204	0.027			
	98	0.173	0.181	0.178	0.043			
	112	0.162	0.181	0.178	0.046			
MCV	0	21.0	21.3	19.8	1.30	0.001	0.001	0.932
(fL)	15	23.7	21.5	19.8	1.32			
	35	25.1	24.9	21.6	2.43			
	49	25.1	26.0	21.9	2.59			
	63	24.1	25.7	21.8	2.39			
	77	25.2	26.2	23.2	2.61			
	98	24.4	26.3	23.0	2.83			
	112	23.3	25.5	23.5	2.53			

RBC, red blood cell; HGB, hemoglobin; HCT, hematocrit; MCV, mean corpuscular volume; T, treatment; SD, standard deviation.

**Table 5:** α-Tocopherol (vitamin E) concentration in the sera of the experimental lambs (*n* = 7).

Item	Day	Control	Herbmix	Selplex	SD	Significance of Effects (*p*-Value)		
						T	Time	T × Time
α-Tocopherol (μg/mL)	0	1.031	1.286	1.164	0.0041	0.001	0.001	0.002
	35	1.035	1.388	1.178	0.6016			
	63	1.079	1.211	1.221	0.2820			
	98	1.417	1.508	1.273	0.4155			

T, treatment; SD, standard deviation.

**Table 6:** Antioxidant status of the experimental lambs (*n* = 7).

Item	Day	Control	Herbmix	Selplex	SD	Significance of Effects (*p*-Value)		
						T	Time	T × Time
GPx	0	412.3	440.6	400.7	66.26	0.001	0.001	0.001
(U/g Hb)	35	420.3	465.9	742.7	159.8			
	63	349.4	384.0	813.2	228.3			
	98	259.6	289.3	795.2	256.4			
MDA	0	0.256	0.275	0.273	0.037	0.132	0.001	0.666
(µmol/L)	35	0.306	0.271	0.301	0.047			
	63	0.230	0.205	0.239	0.039			
	98	0.243	0.207	0.225	0.039			
TAC	0	0.663	0.678	0.690	0.030	0.115	0.001	0.980
(mmol/L)	35	0.667	0.659	0.681	0.036			
	63	0.433	0.429	0.453	0.040			
	98	0.439	0.434	0.449	0.039			

GPx, glutathione peroxidase; Hb, hemoglobin; MDA, malondialdehyde; TAC, total antioxidant capacity; T, treatment; SD, standard deviation.

**Table 7:** Concentrations of thyroid hormones of the experimental lambs (*n* = 7).

	Day	Control	Herbmix	Selplex	SD	Significance of Effects (*p*-Value)		
						T	Time	T × Time
T3 (nmol/L)	35	0.604	1.44	1.57	0.849			
	63	0.529	1.04	1.27	0.784	0.001	0.144	0.021
	98	0.201	0.074	1.96	1.12			
T4 (nmol/L)	35	62.9	72.1	79.7	20.2			
	63	60.2	59.0	69.7	19.0	0.047	0.020	0.849
	98	51.9	46.5	65.4	18.3			
T3:T4	35	0.01	0.02	0.02	0.010			
	63	0.009	0.018	0.018	0.011	0.001	0.230	0.004
	98	0.004	0.002	0.03	0.015			

T3, triiodothyronine; T4, thyroxine; T, treatment; SD, standard deviation.

## Data Availability

Data availability upon reasonable request to the corresponding author.
